# Promotion of osteogenic differentiation by non-thermal biocompatible plasma treated chitosan scaffold

**DOI:** 10.1038/s41598-019-40371-6

**Published:** 2019-03-06

**Authors:** Ying Li, Ji Hye Kim, Eun Ha Choi, Ihn Han

**Affiliations:** 1 Department of Plasma-Bio Display, Kwangwoon University, 447-1, Seoul, 01897 Republic of Korea; 2 Plasma Bioscience Research Center, Applied Plasma Medicine Center, Kwangwoon University, 447-1, Seoul, 01897 Republic of Korea; 3 Department of Convergent manufacturing Engineering, Dankook University, 152, Yongin-si, Gyeonggi-do, 16890 Republic of Korea; 4 Department of Electronic and Biological Physics, Kwangwoon University, 447-1, Seoul, 01897 Republic of Korea

## Abstract

Non-thermal biocompatible plasma (NBP) has recently emerged as an attractive tool for surface modification of biomaterials in tissue engineering. Three dimensional chitosan scaffolds have been widely used in bone tissue engineering due to biodegradable and biocompatible properties. The present study aimed to evaluate osteogenic potential of NBP treated chitosan scaffold. The surface characteristics of scaffolds were analyzed by scanning electron microscopy (SEM) and X-ray diffraction (XRD), cell proliferation and differentiation was tested with osteoprogenitor cell line MC3T3-E1. The results show that NBP modified scaffold increase cell metabolic by MTT assay and live/dead assay. More importantly, we evidenced enhancement of osteogenic differentiation on NBP treated scaffolds by an increase of alkaline phosphatase (ALP) activity, high degree of extracellular mineralization and up-regulated osteogenic marker genes expression level. The findings in our study highlighted NBP as the innovative method to modified chitosan scaffold and to fine-tuning the scaffold a more suitable and beneficial biomaterial for *in vivo* bone tissue engineering and clinical bone defects therapies.

## Introduction

Within recent years, non-thermal biocompatible plasma (NBP) has focused on the development of biological and medical application. NBP was generated by ionizing working gas, composes of a variety of charged particles, reactive oxygen species (ROS), reactive nitrogen species (RNS), ultraviolet light, as well as electric and magnetic field^1^. ROS and RNS has been investigated as the main role in many applications of NBP such as cancer therapy, wound healing, skin diseases, dental care, and sterilization^2–10^. In tissue engineering field, despite of the arising study of NBP acting directly with stem cells or progenitor cells to enhancing differentiation^11–15^, on the other hand, NBP has been extensively studied for surface modification of biomaterial, which is another key element in bone tissue engineering^16–18^.

NBP based strategies as a novel arising method for surface modification of biodegradable polymers, and biomaterials demonstrate great potential. Typically, the consequences of NBP treatment of a material surface include increasing of surface hydropholicity, introducing functional groups to the surface and change the surface roughness^19^. The surface modification process also has shown as gas dependent manner. Inert gas (argon) incorporated oxygen-containing polar functional groups the poly(vinyl alcohol) PVA/chitosan surface whereas reactive gas (oxygen) increased surface roughness leading to improvement of surface wettability^20^. Similar results was reported by Choi *et al*.^18^, NBP treatment increased surface hydrophilicity which using nitrogen and air gas and resulting better osteoblast attachment and proliferation. Lauriault *et al*.^21^ demonstrated NBP induced miropatterned nitrogen-rich depositions to polymer film surface with enhancement of macrophages and chondrocytes adhesion and proliferation and can be used for tissue engineering. These evidence highlighted NBP as a useful tool for surface modification of biomaterial in tissue engineering.

Three-dimensional (3D) scaffolds desired special properties like biodegradable, porosity, mechanical strength and surface chemistry to provide stem cells attachment, proliferation and differentiation in bone tissue engineering. Scaffolds consisting of natural polymers have attracted significant interest due to their flexibility in terms of chemical modification and the ability of degradation into low molecular weight fragments that can be resorbed or eliminated inside human body^22^. Among those chitosan is an abundant natural polyheterosaccharide obtained from deacetylation of chitin^23^. The biological properties of chitosan, including biodegradability, biocompatibility and no toxicity has been well-established presently under extensive investigations for bone regeneration. The improved osteogenic differentiation of stem cells was observed on chitosan scaffold, and *in vivo* study was also conducted^24,25^. The pervasive use of chitosan scaffold in bone tissue engineering has directed much effort towards the advancement of chitosan-base biomaterials through surface modifications or combination with other materials in order to reach higher biocompatibility and osteocondictivity. However, the biological effects, particularly osteogenic differentiation potential of NBP treated 3D chitosan scaffold have not been well studied.

In the current study, we applied NBP treatment to 3D porous chitosan scaffold and evaluated the osteogenic potential of NBP modified chitosan scaffold using MC3T3-E1 cells. For that, we assess cell viability, metabolic ability and particularly osteogenic differentiation by detection of alkaline phosphatase activity, extracellular mineralization and osteogenic marker gene expression of osteoprogenitor MC3T3-E1 cells on NBP treated scaffolds aimed to be used in bone tissue engineering field.

## Results

### Characteristic of reactive species containing in NBP

**Figure 1:**
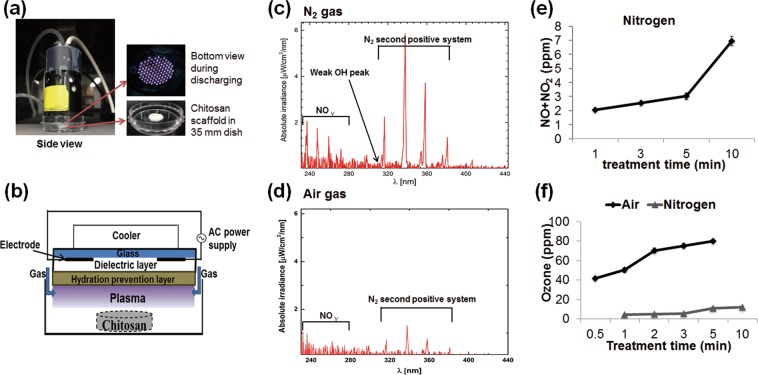
Experimental setup and characteristic of NBP device. (**a**) Experiment setup from side view (left panel) and generation of NBP from bottom view (upright panel) and chitosan scaffold put in the middle of 35 mm dish (downright panel). (**b**) Schematic diagram of dielectric barrier discharge (DBD) plasma device which used to generate NBP. Measurement of optical emission spectra (OES) of plasma generated using (**c**) nitrogen gas and (**d**) air gas. (**e**) Nitric oxides (NO and NO_2_) concentration in NBP during various time discharging using N_2_ gas. (**f**) Ozone concentration in NBP during various time discharging using N_2_ and air gas.

The reactive species containing in NBP was measured by the optical emission spectra (OES). Discharging with N_2_ gas achieved higher NO_γ_ signals (240–280 nm) and N_2_ emission from the second positive (300–400 nm), and weak hydroxyl radical (OH) around 309 nm^26^, as compared with air gas (Fig. 1(c,d)). Furthermore, nitrogen oxides (nitric oxide (NO) and nitrogen dioxide (NO_2_)) accumulation was gradually increasing in a time dependent manner with NBP discharging using nitrogen (N_2_) gas (Fig. 1(e)). However, the ozone (O_3_) accumulation was almost 10 times more when air was working gas than N_2_ was used (Fig. 1(f)).

### Characterization of chitosan scaffold after NBP treatment

**Figure 2:**
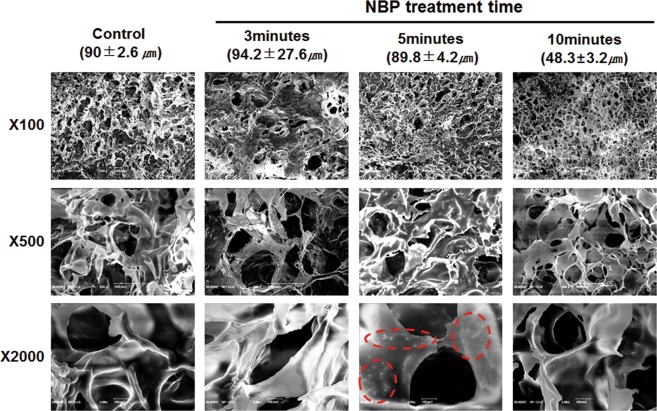
Scanning electron microscopy (SEM) morphology of the 3D chitosan scaffold after NBP treatment. The chitosan scaffolds were treated with NBP for 3, 5, and 10 min, non-treated as control. The average pore sizes (μm) were presented by mean ± standard deviation for each group. SEM images were showed as X100, X500 and X2000 magnifications. The red dotted circle showed newly generated “nanoparticle” structural protrusions on pore barriers after NBP treatment.

Scanning electron microscopy (SEM) micrographs of chitosan scaffolds presented as a 3D highly porous sponge-like microstructure with high degree of interconnection throughout the scaffold in all directions. 5 min treated scaffold showed compactness microstructure with homogeneous pore distribution, and thickened barriers between pores (Fig. 2). Particularly, newly generated surface protrusions were found on the barriers, suggesting an increase of surface roughness by 5 min NBP treatment. However, 3 min scaffold showed heterogeneous pore sizes, and 10 min treatment dramatically decreased the pore size with shrinkage and collapse of pore barriers. However, there was no thermal effect observed on the scaffolds even after 10 min NBP treatment, indicating that our DBD device is suitable for modification on the chitosan scaffold without thermal effect.

**Figure 3:**
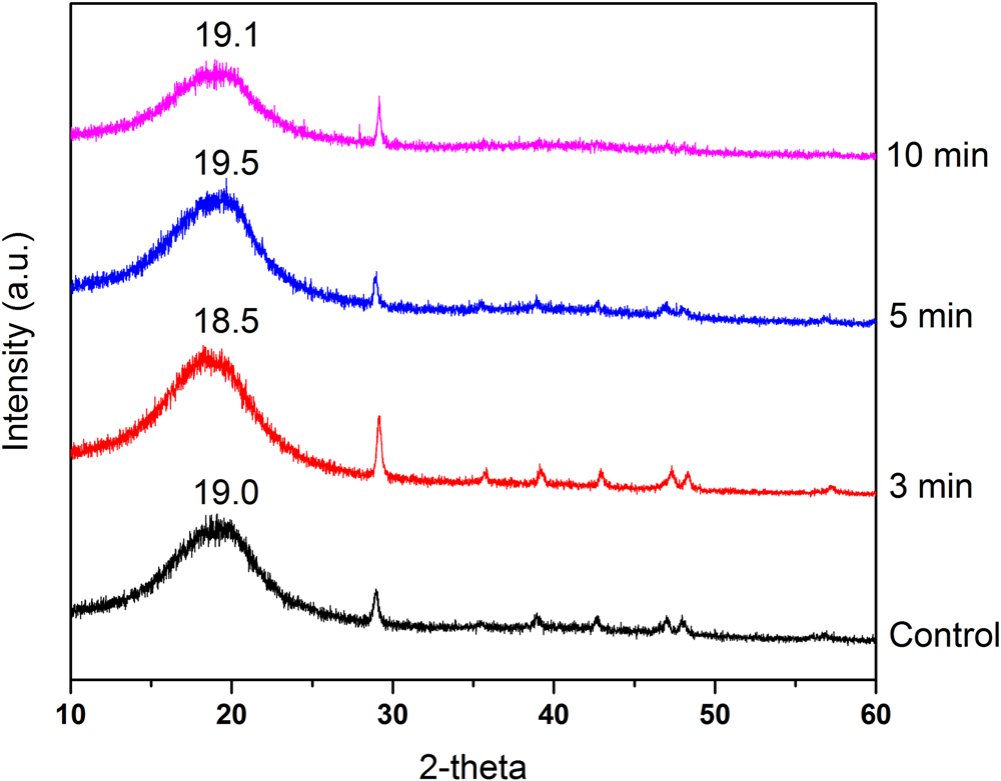
XRD pattern of control, 3 min, 5 min and 10 min NBP treated groups, respectively. The typical fingerprints of semi-crystalline chitosan was exhibited as broad diffraction peaks at 2θ around 19 ° was observed in all groups.

The crystallinity of chitosan scaffold was analyzed using X-ray diffraction (XRD). Figure 3 showed a typical semi-crystallinity with a very broad peak at around 2 θ = 20° of both control and NBP treated scaffolds^23^, suggesting that NBP modification did not change the crystallinity of scaffolds. Whereas, the crystalline size was further calculated by Scherrer equation. The results showed that NBP treated groups the crystallite size reduced 22%, 51%, and 25% for 3, 5, and 10 min treatment, respectively (Table 2). Specially, the 5 min treated significantly decreased the crystallite size by 51%, which is consistent with the SEM morphology analysis that “nano-particle” like deposition on the scaffold pore barriers with NBP treatment of 5 min.

**Table 1:** Technical data of DBD device discharging with N_2_ and air gas.

Parameters	Nitrogen gas	Air gas
Gas flow (liter per min)	1.5	1.5
Voltage (V_rms_, kV)	0.88	0.92
Current (I_rms_, mA)	7.61	7.60
Applied period (µs)	28.50	28.50
Applied frequency (kHz)	35.09	35.09
Plasma on/off-time (ms)	25/150	25/150
Duty ratio (%)	14.3	14.3
Energy per second (J/sec)	0.16	0.14
Energy per second with duty ratio (J/sec)	0.023	0.020

### NBP enhanced cell viability of MC3T3-E1 with chitosan scaffold

**Figure 4:**
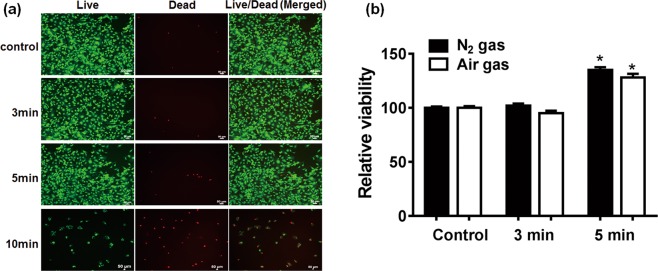
Effect of NBP on MC3T3-E1 cell viability with chitosan scaffolds. (**a**) Live/dead staining to show cell viability on scaffold of control and 3, 5, 10 min NBP treated groups. Green fluorescence represented live cells and red fluorescence indicated dead cells. (**b**) MTS assay was used to detect cell metabolic activity on 3 and 5 min NBP treated scaffold with N_2_ or air gas, untreated scaffold as control. The results are presented as mean ± SD from the three independent experiments. Significant differences were indicated as *P < 0.05.

Cell viability on scaffolds is essential for cellular compatibility and suitability in tissue engineering. As shown in Fig. 4(a), majority of live cells presented as green fluorescence on control (untreated), 3 and 5 min NBP treated scaffold with occasional dead or apoptotic cells (in red fluorescence) indicating no cytotoxicity of scaffold until 5 min NBP treatment. However, 10 min treated scaffold not suitable for cell attachment and survival, as shown by extensive number of dead cells with red fluorescence, which is in consist with scaffold morphology change. Furthermore, cell metabolic ability on a scaffold treated with NBP 5 min shown a significant increase in both N_2_ gas and air gas treatment (*P* < 0.05) (Fig. 4(b)). We did not observe significant differences on cell viability between N_2_ and air gas treatment although there was a big difference of O_3_ level between those two gases. This is because NBP was directly treated on scaffold first, and then the modified scaffold further effects cellular behavior. Considering air discharging produce high level of O_3_, we used N_2_ gas in the following treatment on scaffolds.

### Enhanced osteogenic differentiation of MC3T3-E1 on NBP treated scaffold

**Figure 5:**
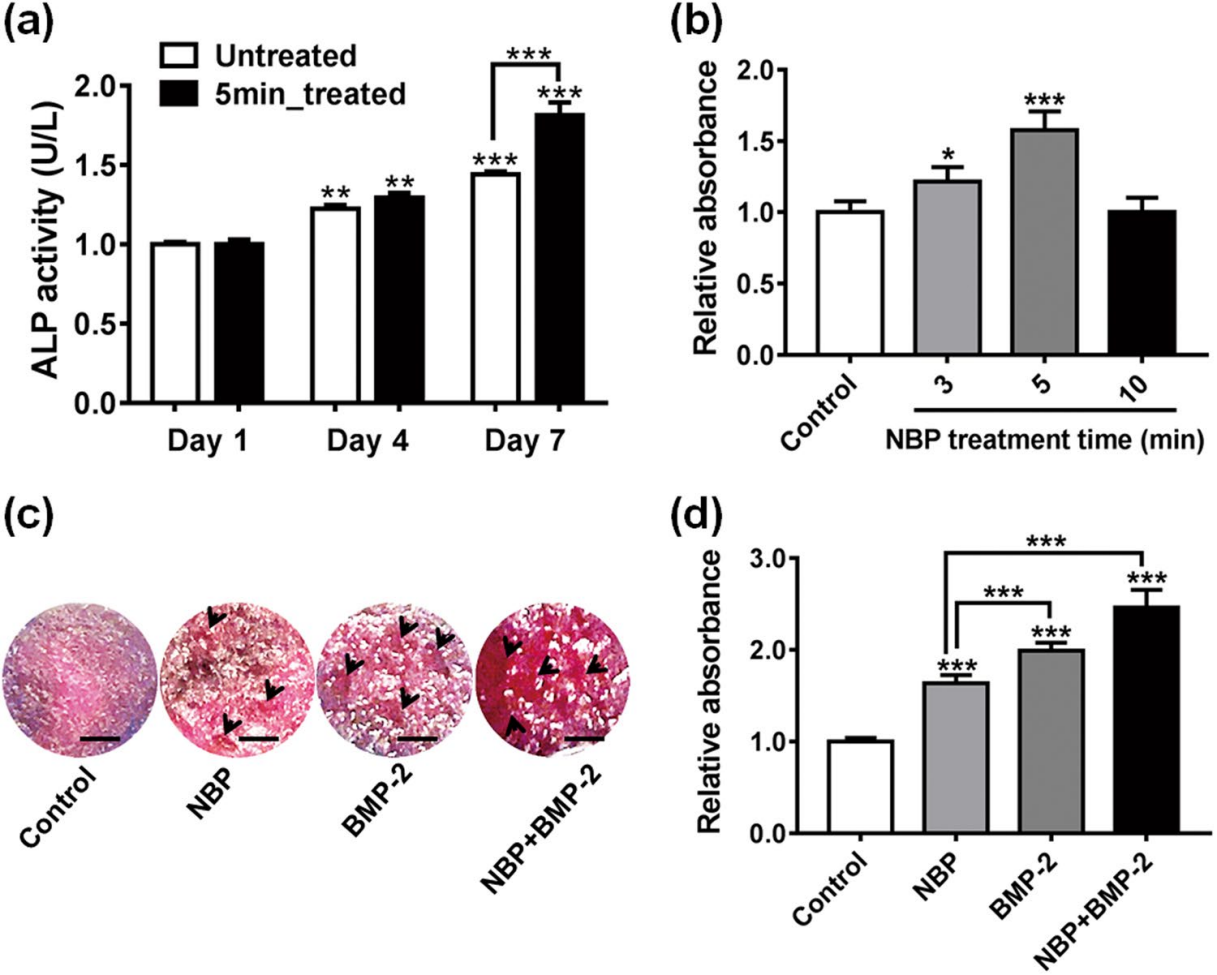
Promotion the osteogenic differentiation of MC3T3-E1 cells by NBP treated scaffold. (**a**) ALP activity assay of MC3T3-E1 cells seeding on untreated and 5 min NBP treated scaffold was performed at days 1, 4, and 7. (**b**) Detection of mineralization on MC3T3-E1 on untreated (control) or 3, 5 and 10 min NBP treated scaffolds at day 4 by ARS staining. Quantitation of mineral deposition by detecting absorbance of ARS extracts. The data are presented as ratio to control. (**c**) ARS staining of MC3T3-E1 cells culture for 4 days on scaffold with or without NBP treatment, and the culture media was supplemented with or without BMP-2. The black arrows showed calcium deposition combined together with ARS staining. Scale bar = 100 μm. (**d**) Quantitation of mineral deposition by detecting absorbance of ARS extracts. Significant differences were indicated as *P < 0.05, **P < 0.01 and ***P < 0.001 by Turkey, on-way ANOVA. Data presented as mean ± SD from three independent experiments.

Alkaline phosphatase (ALP) activity is a well-defined osteogenic differentiation marker and is assumed to reflect the degree of differentiation. In this study, ALP activity of MC3T3-E1 cells on untreated and 5 min NBP treated scaffold was measured at 1, 4, and 7 days. The results showed that ALP activity of cells on both scaffolds gradually increased from day 1 to day 7 (Fig. 5(a)). No significant different between two scaffolds until day4, however, up to 7 days culture, ALP activity was significantly increased in the 5 min treated group than control group (*P* < 0.05).

The formation of calcified nodules is a prominent marker of osteoblast maturation. A confirmation to the mineralization of MC3T3-E1 was examined through alizarin red s (ARS) staining where the binding of calcium ions in mineralized extracellular matrix (ECM) forms an ARS-calcium complex in a chelation process. When compared to original scaffold, NBP 3 (*P* < 0.05) and 5 min (*P* < 0.001) modified scaffold significantly enhanced the formation of calcified nodules in MC3T3-E1 cells after 4 days under osteogenic inductive condition (Fig. 5(b)). Figure 5(c) shows the images of calcified nodules appeared in bright red color on NBP modified scaffold, as the effect of NBP modification alone, bone morphogenetic protein 2 (BMP-2) supplement alone and combined effect of NBP and BMP-2 treatment, confirming the enhancement of calcium nodules on NBP modified scaffold. The quantitative evaluation of cell mineralization revealed the combination effect of NBP and BMP-2 treatment enhanced by 2.5 folds, whereas single treatment increased 1.6 and 2.0 folds, respectively, as compared with control (Fig. 5(d)).

### Upregulation of osteogenic genes of MC3T3-E1 on NBP treated scaffold

**Figure 6:**
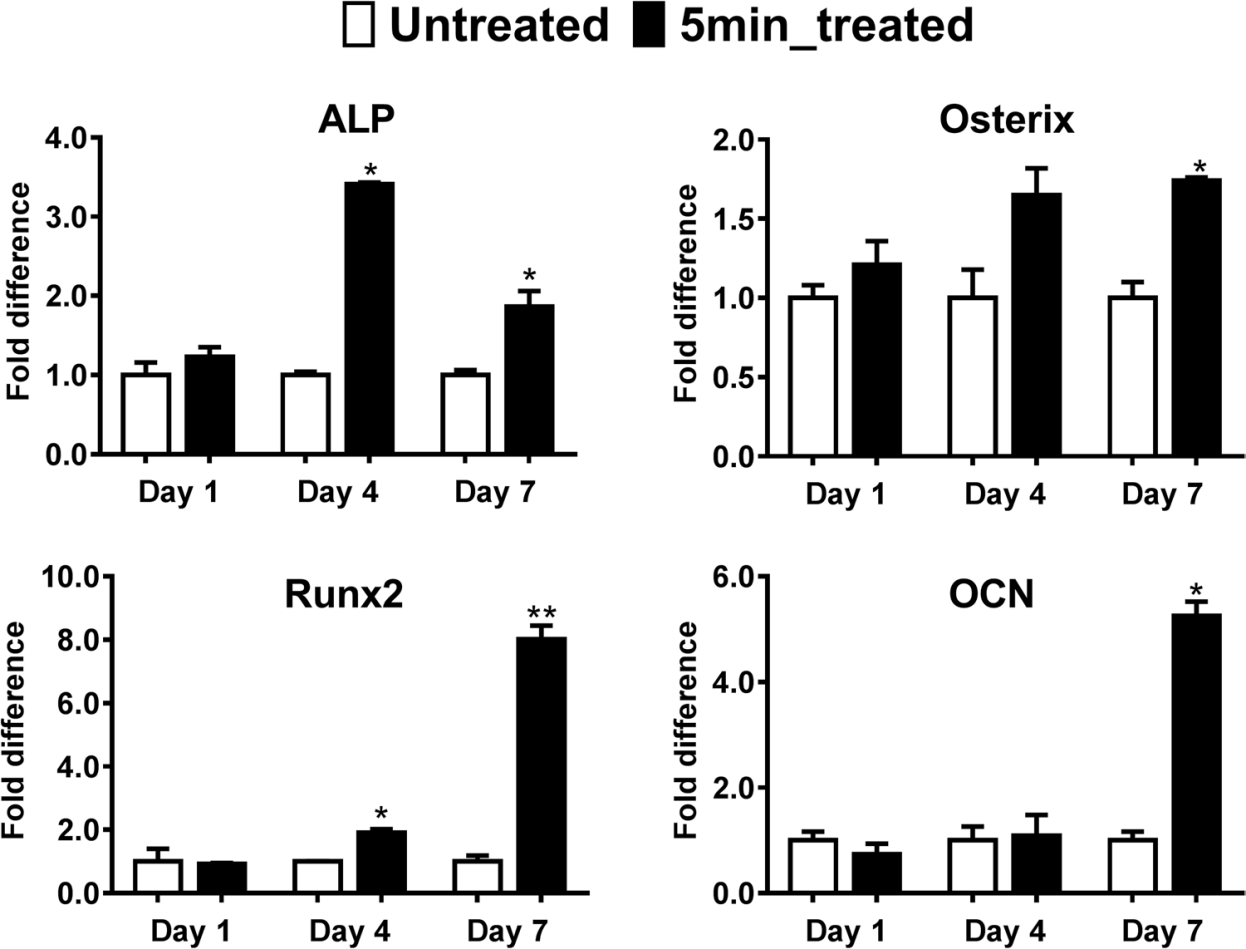
Upregulation of osteogenic genes expression on NBP treated chitosan scaffolds. Osteogenic marker genes (ALP, Osterix, Runx2 and OCN) expression in MC3T3-E1 cells seeding on untreated and 5 min NBP treated scaffolds were examined by a real-time PCR analysis at day 1, 4 and 7, respectively. The housekeeping gene GAPDH was used as a control for the PCR reaction. The results are presented as mean ± SD from the three independent experiments. Significant differences are indicated as *P < 0.05, **P < 0.01.

In order to explore the differentiation effect of NBP treated scaffold on MC3T3-E1 cells, expression of osteogenesis related genes was analyzed by real-time PCR. As shown in Fig. 6, ALP and Runt-related transcription factor 2 (Runx2) showed significant upregulation on 5 min NBP treated scaffold than untreated scaffold at day 4 (*P* < 0.05), and maintaining the high level until day 7. Osterix expression exhibited significant increase at day 7 (*P* < 0.05). As a late stage maker, osteocalcin (OCN) was significantly upregulated at 7 days culture.

### Energy-dispersive X-ray spectroscopy (EDS) analysis for extracellular mineralization

EDS analysis was used to analyze the mineralization in the osteogenic differentiation of the calcium and phosphate deposits in the extracellular matrix. Quantitative elemental EDS analyses showed that the amount of calcium deposition increased after 7 days incubation, and significantly increased in the 5 min NBP treated chitosan scaffolds group (Table 3). However, a similar pattern was not observed in the phosphorus deposition. The experimental ratio of Ca/P showed no difference between control and 5 min treated group on day 1 and day 4, whereas at day 7 the treated group is higher than control, which is consistent with the ALP activity results (Fig. 5(a)). However, this Ca/P ratio indicated only within single cells, rather than the whole bone structure Ca/P ratio, which can be used for analysis of cellular behavior during osteogenic differentiation.

**Table 2:** Crystallite size and percentage change in crystallite size of control and NBP treated chitosan scaffold (“—” indicated decreasing of crystallite size).

Treatment	Crystallite size (nm)	% change in crystallite size
control	1.42	—
3 min	0.78	−22%
5 min	0.69	−51%
10 min	0.75	−25%

## Discussion

NBP exists as a quasi-neutral particle system in the form of gaseous or fluid-like mixtures of free electrons, ions, and radicals, generally also containing neutral atoms and molecules. Some of the neutral particles are in an excited state, which can return to the ground state by photon emission. The detection of spectrum of these emissions by optical fiber can speculate the radical contained in NBP. In the present study, when nitrogen gas is used, OES shows excited N_2_ secondary positive system and NO_γ_ spectrum. These exited species bombarded onto scaffold surface, offering an effective strategy to incorporate reactive functional groups on chitosan scaffold surface.

For chitosan scaffold surface, amino group and hydroxyl group in chitosan provide functionality. The free amino group confers a positive charge to chitosan, allows it for many electrostatic interactions with electrons and negatively charged molecules^22^. So chitosan turns out to be a highly reactive polysaccharide. Electrons and reactive oxygen and nitrogen species generated from NBP interacted with the surface molecules, resulting in increasing number of oxygen- and nitrogen- containing groups and polar groups on the surface, showing with increased the surface roughness with emergence of sharp protuberances of chitosan film^27^. Silva SS *et al*.^28^ has demonstrated that when nitrogen was used as feeding gas, not only the etching process but also functionalization with nitrogen- containing group was observed on NBP modified chitosan membranes, which is consistent with the SEM results in present study. NBP treatment increased the pore size and thickened the pore barriers and resulted in the structural compactness of entire scaffold. The structural compactness change and thickened interconnectivity may due to post NBP treatment effect of surface adaptation and oxidation reactions, leading to an increase of cell affinity to the surface and improving cell viability and proliferation.

Recent studies have indicated that the surface characteristics and microenvironment of scaffolds, including chemical deposition, roughness, hydrophobicity and matrix elasticity, can profoundly affect cell behavior and functioning in directing the osteogenic differentiation of stem cells^29^. Cell viability on scaffolds is essential for cellular compatibility and suitability for tissue engineering applications^19^. In our study, the cell viability significantly increased on the 5 min NBP treated scaffold (*P* < 0.05), indicating that NBP treatment improves cell adhesion and growth on the scaffolds.

Following cell attachment and proliferation on scaffold surface, cells start to differentiate into osteoblast phenotype under osteoinductive media. Three major transformation stages of cell proliferation, maturation, and mineralization of ECM exist during the growth and differentiation of MC3T3-E1 cells to osteoblast lineage, accompanied with up-regulation of certain related proteins and genes of each stage. ALP enzyme indicates early stage of osteoblast activity and maturation. ALP activity in MC3T3-E1 cells on both scaffolds were increased as an incubation time dependent manner, and at day 7 ALP activity was significantly higher on NBP treated scaffold than control scaffold (*P* < 0.001), suggesting that NBP modification of scaffold promote osteogenic differentiation. Mineralization is the final phase of osteoblast differentiation, whereby the mineral matrix, which predominately contains calcium phosphate in the form of hydroxyapatite, is secreted and deposited by mature osteoblasts. Evidence of osteogenic differentiation of MC3T3-E1 cells can be found in their capability to mineralize inorganic calcium at the middle of the differentiation period, monitored as inorganic mineral deposition. In our study, the ARS staining and EDS results demonstrate the beneficial action of the NBP modified chitosan scaffolds on osteoblast ECM mineralization. Besides, BMP-2 plays pivotal role in osteogenic differentiation and bone regeneration^30^. A combined BMP-2 and NBP stimulation augmented mineralization to a greater degree than treatment with either single stimulus, suggesting the combined method *in vitro* could be considered as groundwork for *in vivo* bone development.

Real-time PCR analysis of osteogenic gene expression further indicates that NBP modification to scaffold is favorable for osteogenic differentiation. In this study, gene expression of ALP, runt-related transcription factor 2 (Runx2) was significantly upregulated on NBP modified scaffold at day 4 (*P* < 0.05), whereas osterix showed significant increased upregulation at day 7 (*P* < 0.05), as compared with untreated scaffold. ALP is a well-known marker of osteogenic differentiation for cells at an early stage^31^ and osterix is an osteoblast-specific transcription factor essential for osteogenic differentiation and bone formation. Runx2 can act as an indispensable factor transcriptionally regulating osteocalcin (OCN) and ALP gene expressions, participating in controlling osteoblast function and ECM mineralization in osteogenesis^32^. The higher expression level of ALP, Runx2 and osterix by MC3T3-E1 cultured on NBP modified scaffold indicates that the modification has an enhanced ability to supported cell differentiating into osteoblast phenotype.

The osteogenic potential of scaffold can be explained by surface microenvironment change after NBP treatment. It has been demonstrated that architecture of scaffold surface are able to influence cell behavior. The biological properties of scaffold come from its chemical composition^22^. In our study, SEM results revealed that scaffold with NBP modification increased surface roughness with newly generated nanoparticle-like structure protrusions. Similar pattern was observed in our previous study of NBP treated zirconia, a dental implant material, also increased osteogenic potential after modification^33^. Engler *et al*.^34^ had interpreted that the role of matrix microenvironment directing stem cell differentiation comes at the late stage, while in the early stage the chemical inductors play the major role. This could explain that in our study the ALP enzyme activity in MC3T3-E1 cells presented significant difference at day 7 on NBP modified scaffold, and when adding inducer BMP-2, ostegenic process was further promoted with enhanced mineralization at day 4.

In this study, we found that NBP treated scaffolds has an enhanced ability to support osteoblast phenotype with higher level of ALP activity, mineralization and osteogenic gene expression as compared with untreated scaffolds. Our results proved that NBP modification as a new technique can be used for fine-tuning the scaffold surface and increase its osteogenic potential, providing critical insight into the future use of NBP modified chitosan scaffold as one of the best candidates for *in vivo* study and for the clinical application of bone implant engineering.

## Materials and Methods

### Chitosan scaffold preparation and characteristics

The chitosan scaffolds used in this study were prepared and provided by Dankook University. The preparation of chitosan scaffold was described in previous studies^35,36^. Chitosan solution was dissolved to 1.5 wt% in 0.5 v/v% acetic acid (CHT solution).

CHT solution with mineral oil/Span80 (14:1% v/v, MS solution) was improved dispersion force and physical property. Blend solution (CHT/MS = 60:10% v/v) strong stirring 350 rpm for 1 h after the solutions were injected into the mold (internal diameter 10 mm), frozen at −80 °C for 3 h. 0.5 N NaOH were injected into the mold at 1 °C for 8 h after scaffold was washed in ethanol(100, 80, 60 and 40% v/v) stage by for 5 h each. Finally, after washing for 48 h in distilled water, the scaffold was frozen at −80 °C for 24 h and then lyophilized for 72 h.

### Experiment setup

Figure 1(a) shows the experiment setup of which chitosan scaffolds were treated with NBP in a 35 mm culture dish using a dielectric barrier discharge (DBD) device. The right up picture shows from top view of DBD during discharging, while the right down picture shows a scaffold placed in the center of the dish. The DBD device for generating NBP has been described in details in our previous studies^37,38^. Figure 1(b) is the schematic diagram of DBD device used in this study, with electrodes thickness of 5 μm, electrode gap of 200 μm, and electrodes were covered with dielectric layer of 30 μm in thickness. And hydration protection layer was made of aluminum oxide (Al_2_O_3_) with thickness of 1 μm. NBP was generated with a discharge voltage 500 V and electric current of 13 mA with alternating current (AC) electric power supply. Nitrogen (N_2_) or air gas with a flow rate of 1.5 liter per minute (lpm) was used as working gas. The technical data of DBD device discharging with N_2_ and air was shown in Table 1. Scaffolds were treated with NBP for various time of 3, 5, and 10 min. In Fig. 1(c,d), the OES of DBD discharging with N_2_ and air gas were measured by the spectrometer (HR4000, Ocean Optics, Dunedin, FL). The intensity of optical emission signals was recorded in terms of wavelength (range from 280 to 450 nm). In Fig. 1(e,f) shows the concentration of O_3_ and nitrogen oxides (NO and NO_2_) during various NBP discharging time measured using a Gastec AP-20 gas sampling pump (Gastec Corp., Kitagawa, Japan) and Gastec detector tubes (No. 18 M and 18 L for O_3_, No. 11 L for NO and NO_2_).

**Table 3:** NBP treatment enhanced MC3T3-E1 cells mineralization with chitosan scaffold by EDX analysis.

	Day 1		Day 4		Day 7	
	cont	5 min	cont	5 min	cont	5 min
Calcium (%)	0.02 ± 0.01	0.01 ± 0.01	0.03 ± 0.01	0.02 ± 0.02	5.52 ± 0.58	10.22 ± 0.65
Phosphorus(%)	0.31 ± 0.13	0.61 ± 0.15	0.26 ± 0.17	0.48 ± 0.14	0.53 ± 0.30	0.57 ± 0.09
Ca/P ratio	0.06	0.02	0.12	0.04	10.42	17.93

The calcium and phosphorus percentages were detected after incubation for 1, 4, and 7 days, respectively. The results are presented as mean ± SD from the three independent experiments.

### Scanning electron microscopy observation

The characteristic of original chitosan scaffolds and scaffolds treated with NBP for 3, 5 and 10 min, respectively, were analyzed using SEM. 3 scaffolds of each group were coated with platinuim at 20 milliampere (mA) for 60 sec, and then examined by SEM (Hitachi S-3000N, Japan).

### X-ray diffraction (XRD) study

XRD pattern of 3, 5, and 10 min NBP treated and non-treated chitosan scaffolds was analyzed using Bruker D8 Advance X-ray diffractometer system (Bruker AXS GmbH, Germany) at room temperature. The wavelength of the radiation was 0.154 nm, from a broad focus Cu tube operated at a voltage of 40 kV and a current of 25 mA was applied to the samples for measurement. The data was obtained in the form of 2-theta versus intensity (a.u.) chart. Then the obtained data was used for calculation of crystallite size using the following Scherrer Equation:

$${\rm{Crystallite}}\,{\rm{size}}=k{\rm{\lambda }}/({\rm{FWHM}}\ast {\rm{\cos }}\,{\rm{\theta }})$$

where k is the equipment constant with a value of 0.94, λ is the wavelength of the radiation, θ is the diffraction angle of the peak, FWHM indicated Full Width at Half Maximum of the peaks. Percentage change in crystalline size was calculated using following formula:

$${\rm{Percentage}}\,{\rm{change}}\,{\rm{in}}\,{\rm{crystalline}}\,{\rm{size}}=[({{\rm{G}}}_{{\rm{t}}}-{{\rm{G}}}_{{\rm{c}}})/{{\rm{G}}}_{{\rm{c}}}]\,\times \,\mathrm{100} \% $$

where G_c_ and G_t_ are crystallite size of control and treated samples, respectively.

### Cell culture with chitosan scaffolds

MC3T3-E1 cells from normal mouse osteoblasts were obtained from the American Type Culture Collection (ATCC, Rockville, MD, USA). Cells cultured in α-minimal essential medium (α-MEM; HyClone, USA) containing 10% (v/v) heat inactivated fetal bovine serum (FBS) (Gibco) and 1% (v/v) antibiotics (Gibco) until adequate cell number was obtained. The cells used in the all experiments were less than passage 8. Cells were seeded on the scaffolds (both untreated and NBP treated) placed in a 24-well plate with 100 μl of cell suspension containing of 1 × 10^5^ cells. After 3 h incubation, another 900 μl of culture media was added to each well, and the culture was maintained at 37 °C in 5% CO_2_ and 95% humidity. For osteogenic differentiation assays (ALP activity, ARS staining and osteogenic marker expression), the culture media was changed into osteogenic induction media supplemented with 10% FBS, 10 mM β-glycerophosphate, and 50 µM L-ascorbic acid 2-phosphate (Sigma-Aldrich).

### Live/dead assay

Cell viability was assessed using live/dead assay for original and modified scaffolds. Initially, chitosan scaffolds were treated by NBP for 3, 5 and 10 min. Immediately post treatment, 1 × 10^5^ cells were seeded on each scaffold placed in a 24-well plate. The analysis was conducted for an incubation period of 24 h culture after seeding using a live/dead viability/cytotoxicity kit (Molecular probes, USA) and following the manufacturer’s instructions. Before the live/dead staining, the cell-scaffolds were washed with PBS. 2 μl of 50 μM calcein and 4 μl of 2 mM ethidium homodimer-1 in 1 ml PBS were added to each well and incubated for 20 min at room temperature (RT) in the dark. Images of the cells were then captured under a fluorescence microscope (Nikon, USA).

### Cell metabolic activity on scaffolds

[3-(4,5-dimethylthiazol-2-yl)-5-(3-carboxymethoxyphemyl)-2-(4-sulfophynyl)-2H-tetrazolium] (MTS) assay was performed on cells seeded on original and NBP 3 and 5 min treated scaffolds. MTS was used as a reagent to detect cell metabolic activity. The metabolically active dehydrogenase enzymes in the cells reduce MTS to soluble formazan compounds. Absorbance was detected at 490 nm using a microplate reader (Biotek, VT, USA). The results were present as relative viability (% to control).

### Measuring ALP activity

The effect of NBP on the ALP activity of the MC3T3-E1 cells was analyzed using an ALP assay kit (BioVision, USA). The MC3T3-E1 cells were seeded on non-treated and 5 min NBP treated chitosan scaffolds with culture media. After 24 h incubation, the medium was changed to osteogenic induction medium and cultured for 1, 4 and 7 days. At each time point, ALP activity was determined in the cell supernatant following assay kit protocol. The absorbance was read at 405 nm in a microplate reader (Gen5), and calculated according to the p-nitrophenol (pNP) standard curve. ALP activity (U/ml) = A/V/T (A: amount of pNP generated by samples (µmol), V: volume of sample added to the assay well (ml), T: reaction time (min)).

### Determination of mineralized matrix

Mineralization in MC3T3-E1 cells on NBP modified scaffolds was determined by staining with ARS, which selectively binds with calcium and yields a bright red staining. Cells were seeded at a density of 1 × 10^5^ cells on original and 5 min NBP treated chitosan scaffold with culture media. 24 h post incubation, the medium was changed to osteogenic induction media, and incubated for 4 days. The cells were rinsed twice with PBS and fixed with 4% paraformaldehyde for 10 min, followed by staining with 2% ARS solution (pH 4.2) for 1 h at room temperature. After aspiration of the unincorporated dye, the wells were washed 4 times with PBS. For quantification of calcified matrix, 1 ml of 10% (v/v) acetic acid was added to each well, and the plate was incubated at room temperature for 30 min with shaking. Aliquots (100 µl) of the supernatant were read at 405 nm in 96-well using a microplate reader (Gen5).

Further ARS staining and quantitation was performed to MC3T3-E1 cells seeded on untreated scaffold (with or without BMP-2 supplement) and 5 min NBP treated scaffold (with or without BMP-2 supplement), respectively. BMP-2 was supplemented in culture media at the concentration of 200 ng/ml and cells were cultured for 4 days. Similar process was followed for ARS staining and absorbance reading. The images of calcium deposition on scaffolds were photographed using a camera.

### Quantitative real-time polymerase chain reaction (qRT-PCR)

Expressions of osteogenic marker genes were quantified by quantitative real-time polymerase chain reaction (qRT-PCR) after ribonucleic acid (RNA) isolation and complementary deoxyribonucleic acid (cDNA) synthesis. Total RNA was isolated from the MC3T3-E1 cells using the RNeasy Mini Kit (Qiagen, Inc., Texas, USA). cDNA was synthesized using the superscript VILO cDNA synthese kit (Invitrogen, USA) following the manufacturer’s instructions. 4 µl of each cDNA was used in the PCR reactions (iQ SYBR® Green Supermix kit, Bio-Rad Laboratories, Richmond, CA, USA). The amplifications were performed using the following protocol: 40 cycles of 95 °C for 15 s and 60 °C for 60 s. The primers included ALP, Osterix, Runx2, OCN (Bionix, Seoul, Korea). The primer sequences were shown in Table 4. *Glyceraldehyde 3-phosphate dehydrogenase* (GAPDH) was used as the control gene to normalize RNA expression. All reactions were run in triplicate for three independent experiments.

**Table 4:** The primer sequences for quantitative real-time polymerase chain reaction (qRT-PCR).

Osteogenic marker	Forward primer	Reverse primer
*ALP*	ATCTTTGGTCTGGCTCCCATG	TTTCCCGTTCACCGTCCAC
*Runx2*	AAATGCCTCCGCTGTTATGAA	GCTCCGGCCCACAAATCT
*Osterix*	AGCGACCACTTGAGCAAACAT	GCGGCTGATTGGCTTCTTCT
*OCN*	CCTGAGTCTGACAAAGCCTTCA	GCCGGAGTCTGTTCACTACCTT

### EDS analysis

MC3T3-E1 cells were seeded on the non-treated and 5 minutes NBP treated chitosan scaffolds in general media. After 24 hours incubation, the media was changed to osteogenic induction media to evaluate cell mineralization at 3 time points (1, 4, and 7 days, respectively). At each time point, the chitosan scaffolds with cells were gently rinsed with PBS and primary fixed with Karnovsky’s fixative (2% paraformaldehyde +2% glutaraldehyde) at room temperature for 2 hours. The samples were washed with PBS three times as the secondary fixation with 1% osmium tetroxide (OsO4) at room temperature for 30 min, followed by washing with PBS twice. Alcohol gradient dehydrating was performed to remove water from the scaffolds at increasing ethanol concentrations (30%, 50%, 70%, 80%, 90%, and 100%), and the samples were then dried with hexamethyldisilazane (HMDS) twice (10 minutes each). The samples (n = 3) were coated with platinuim at 20 milliampere (mA) for 60 seconds and analyzed the presence of calcium and phosphorus elements at the surface by EDS (Bruker Nano GmbH, Berlin, Germany).

### Statistical Analysis

Graphs are represented by the mean ± standard deviation of replicates. Student’s *t*-test was used for two groups comparison. One-way analysis of variance was used to compare the results between more than two different groups followed by Turkey’s multiple comparisons test. The statistically significant differences were based on *P < 0.05, **P < 0.01, and ***P < 0.001. All graphing, calculation and statistical analysis were performed using GraphPad Prism version 7.00 (GraphPad Software, USA, www.graphpad.com). All assays involved at least three independent sets of tests for each group scaffolds.
